# Supplementary material for: The mutagenic forces shaping the genomic landscape of lung cancer in never smokers
Source: medRxiv. 2024 May 17:2024.05.15.24307318. Preprint. [Version 1] doi: 10.1101/2024.05.15.24307318 (PMC11118654; doi:10.1101/2024.05.15.24307318)

Extended Data Fig. 1

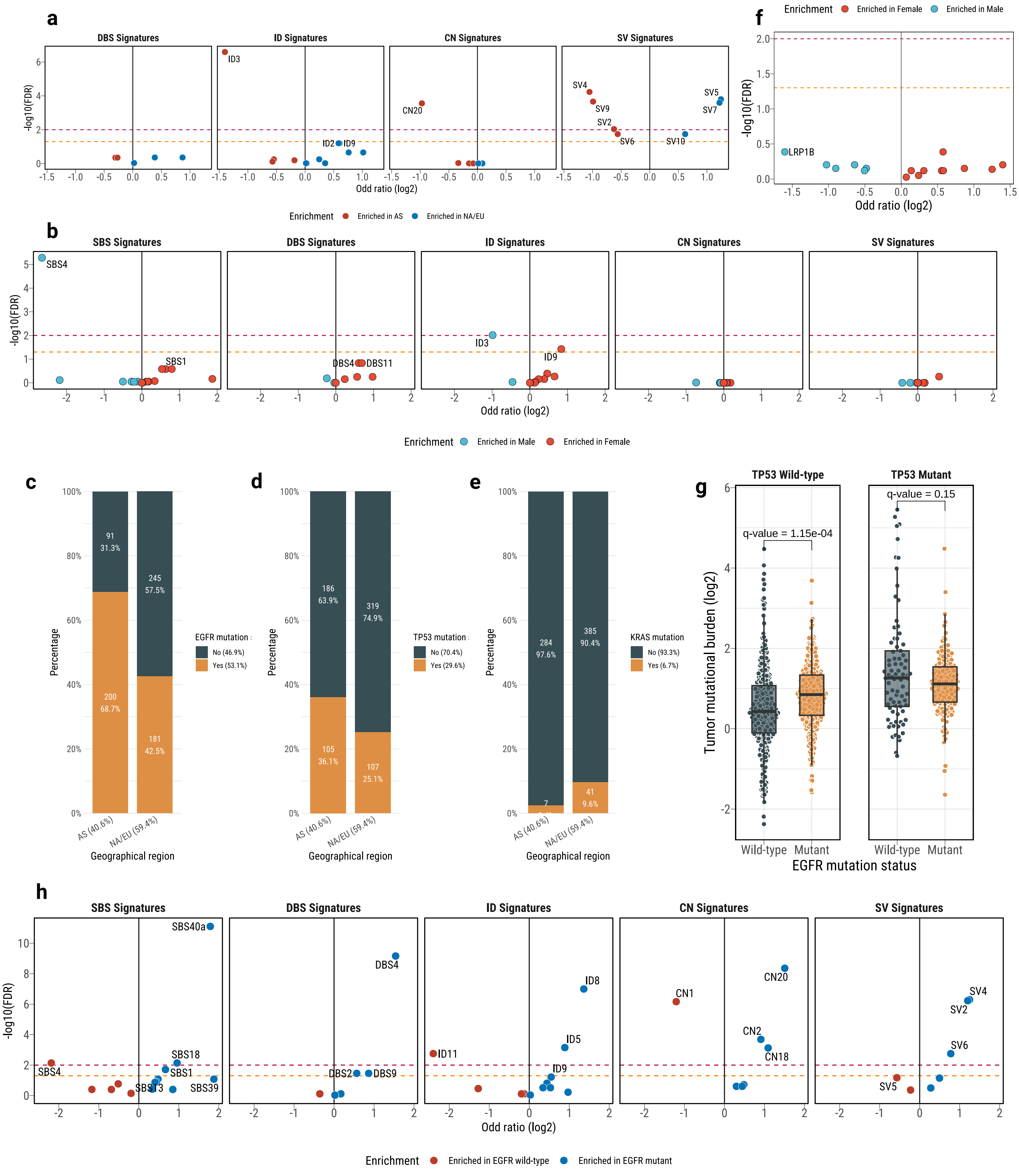

Extended Data Fig. 2

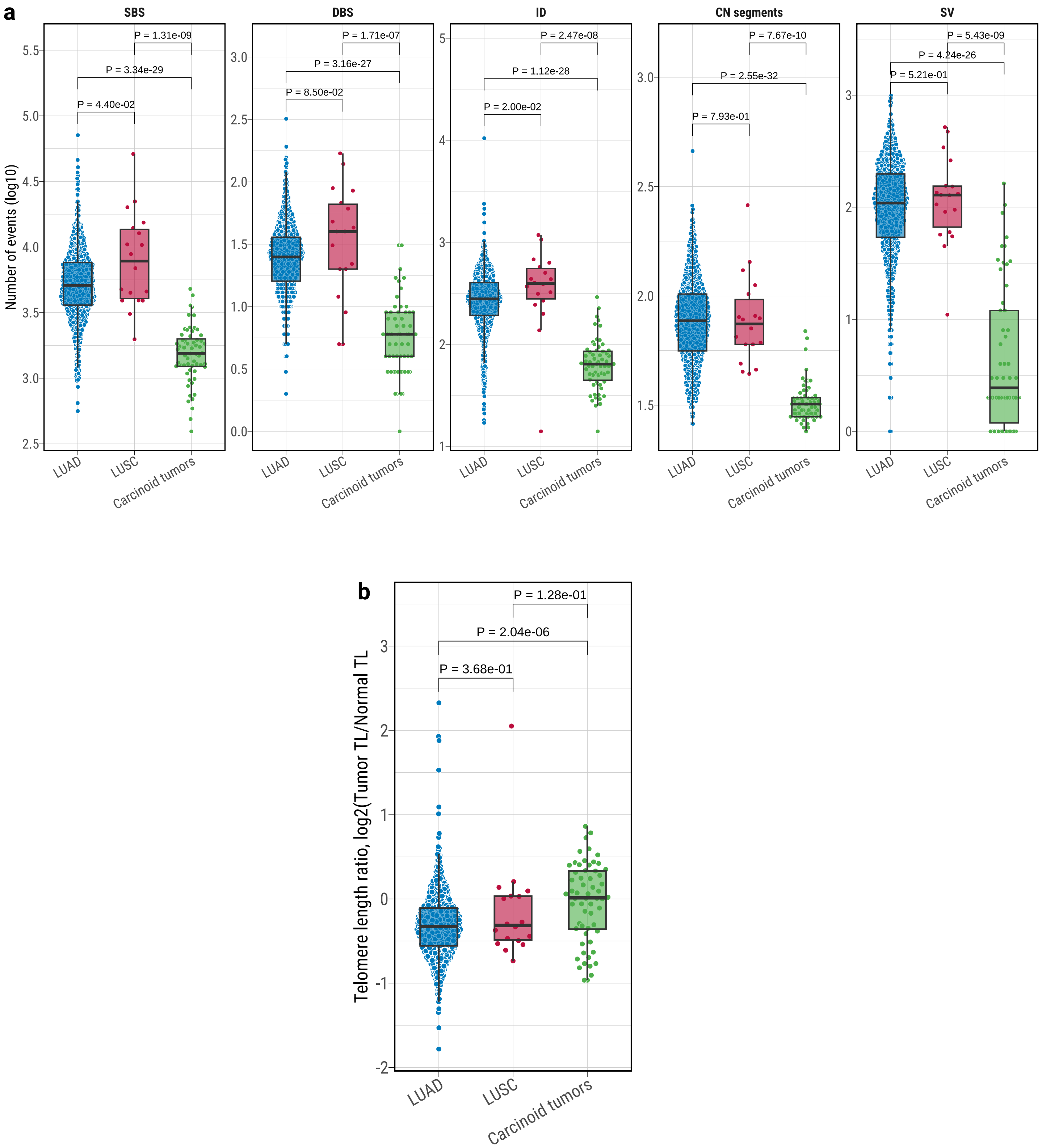

Extended Data Fig. 3

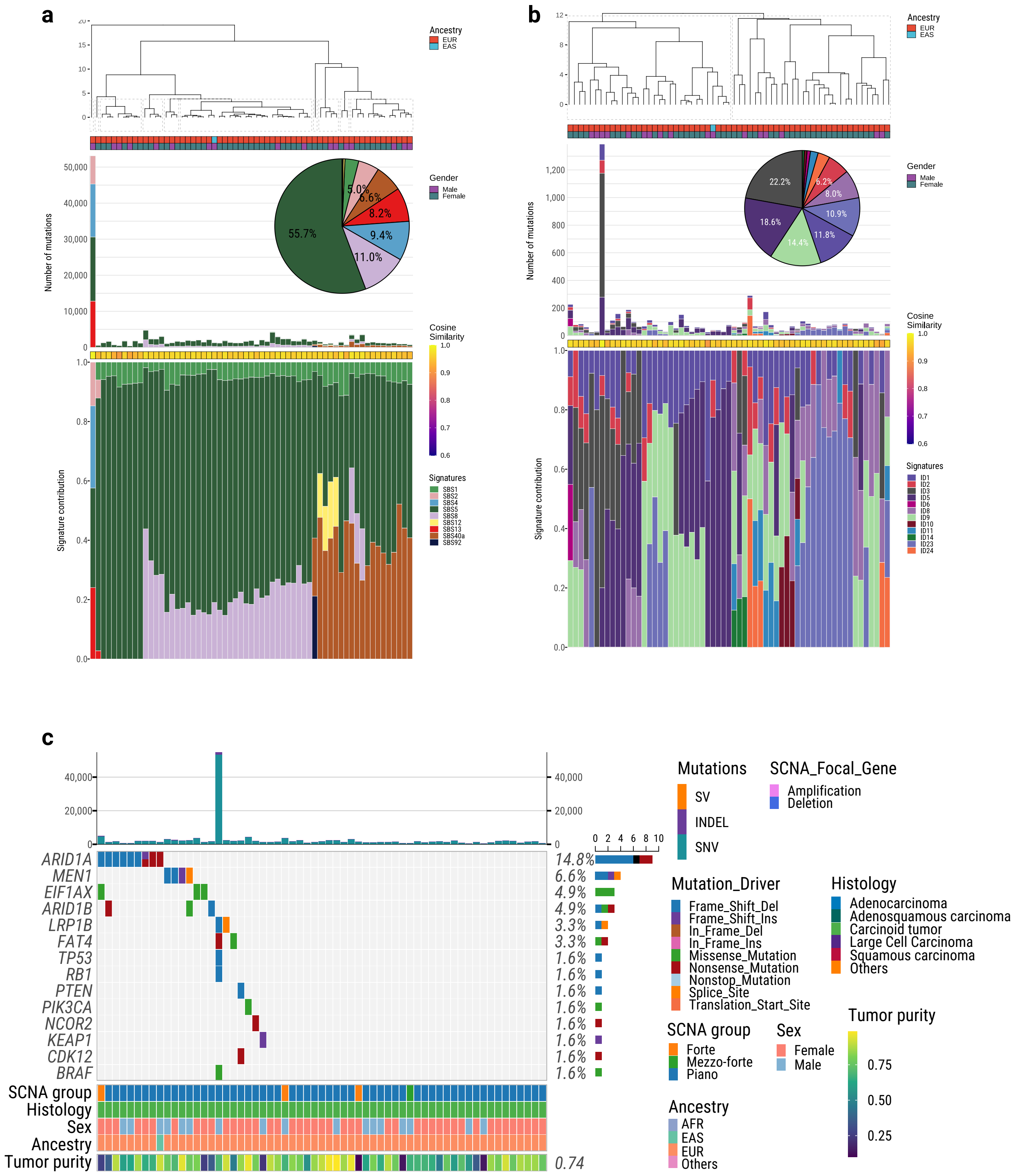

Extended Data Fig. 4

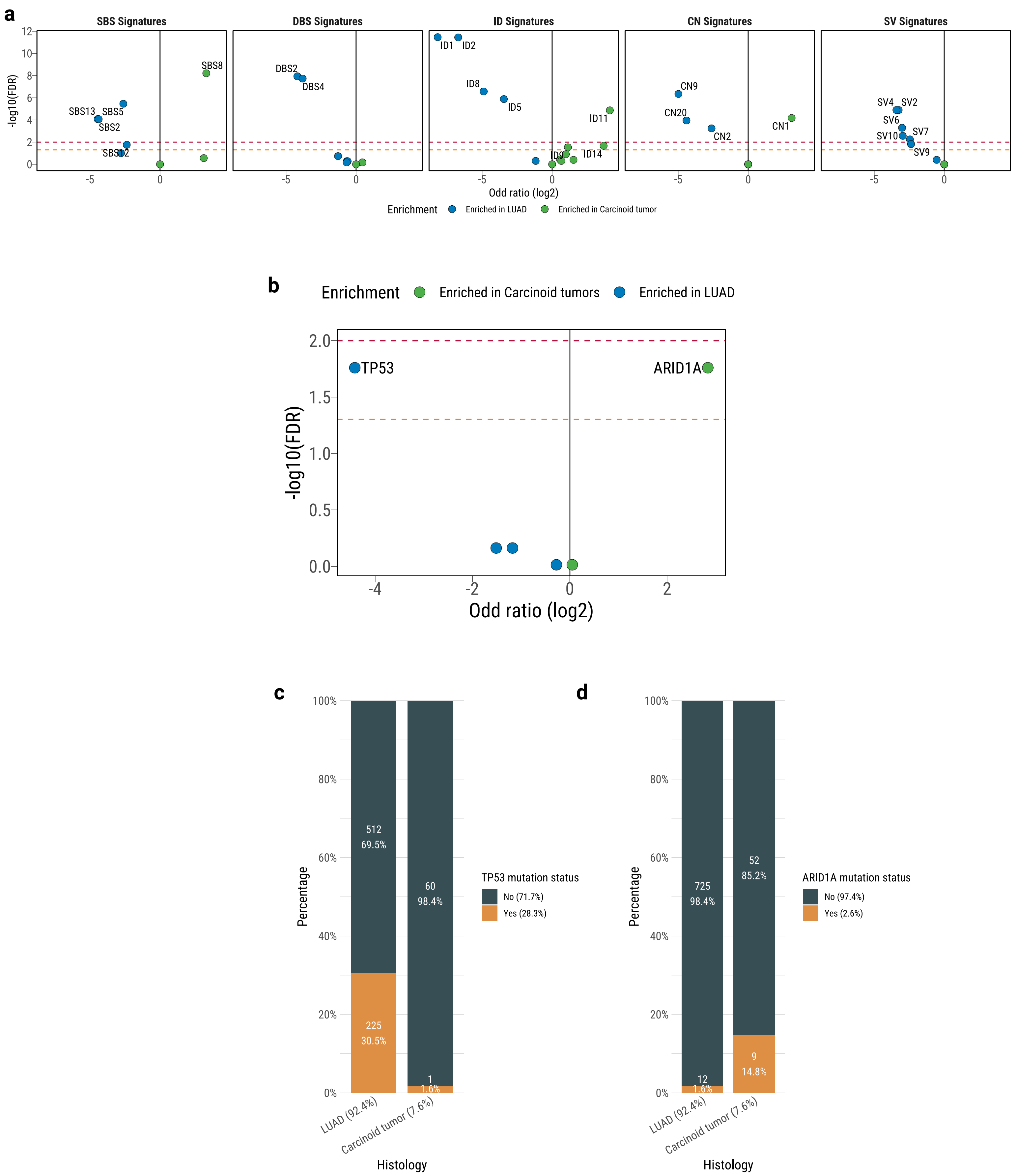

Extended Data Fig. 5

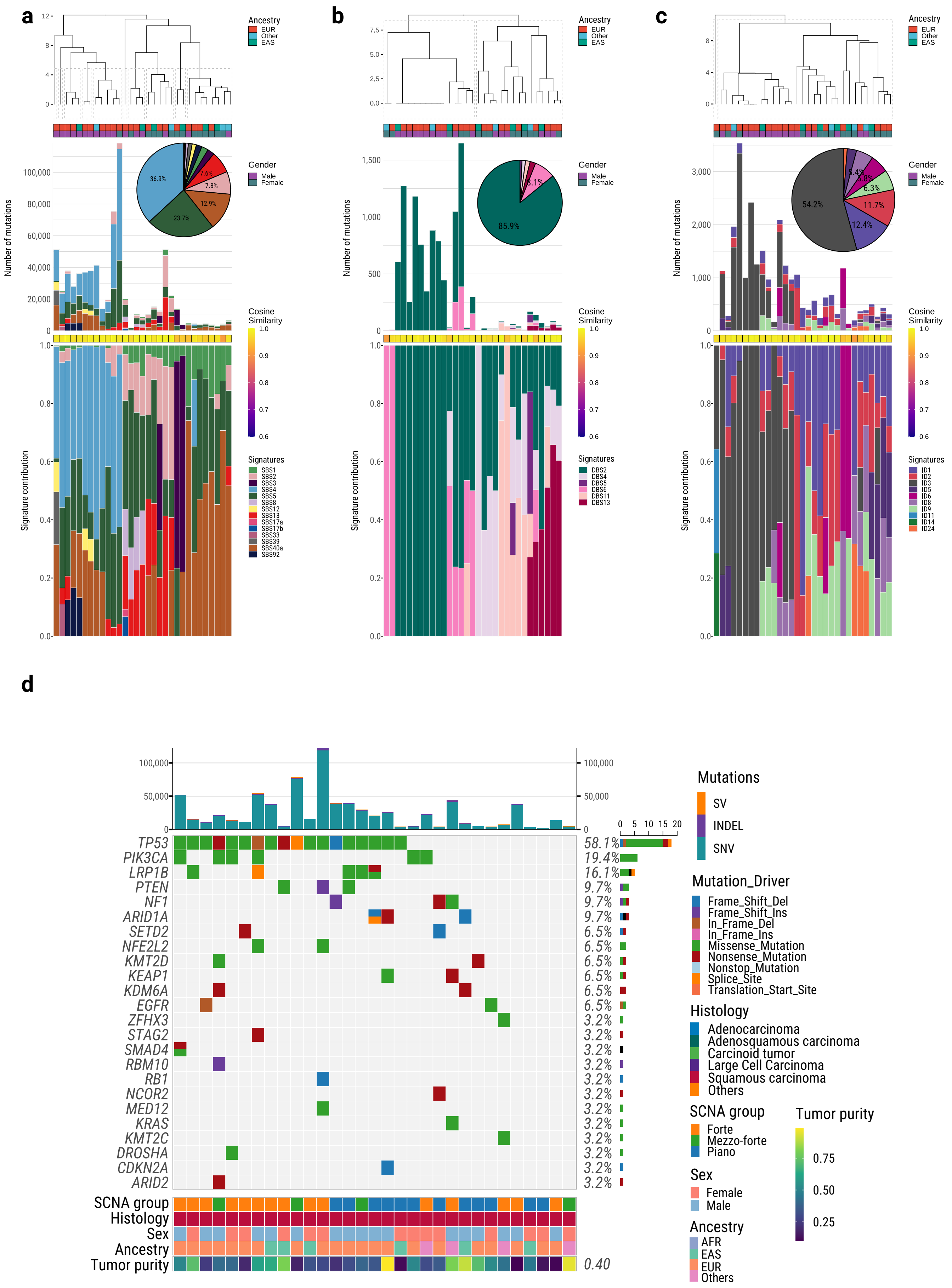

Extended Data Fig. 6

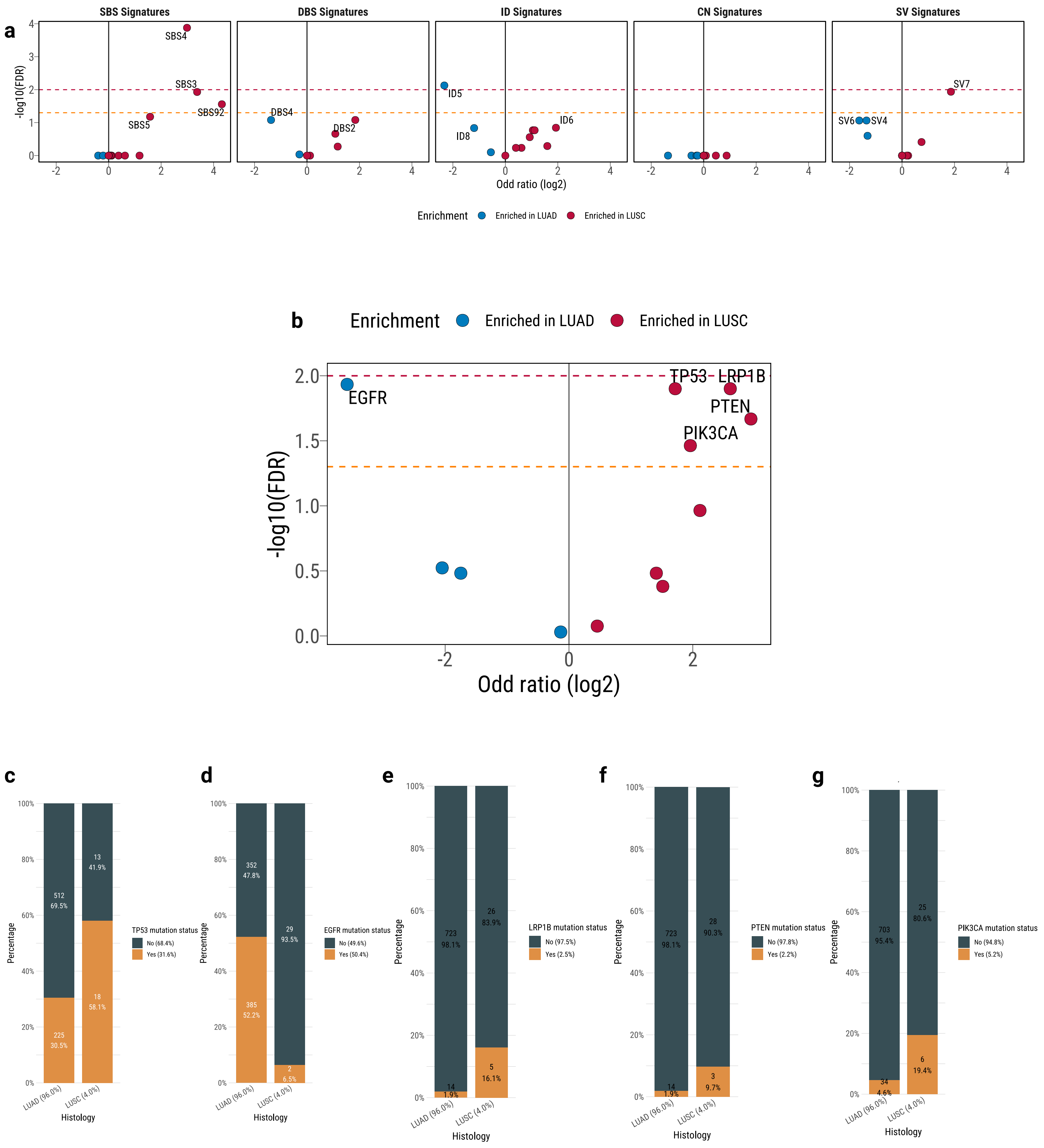

Extended Data Fig. 7

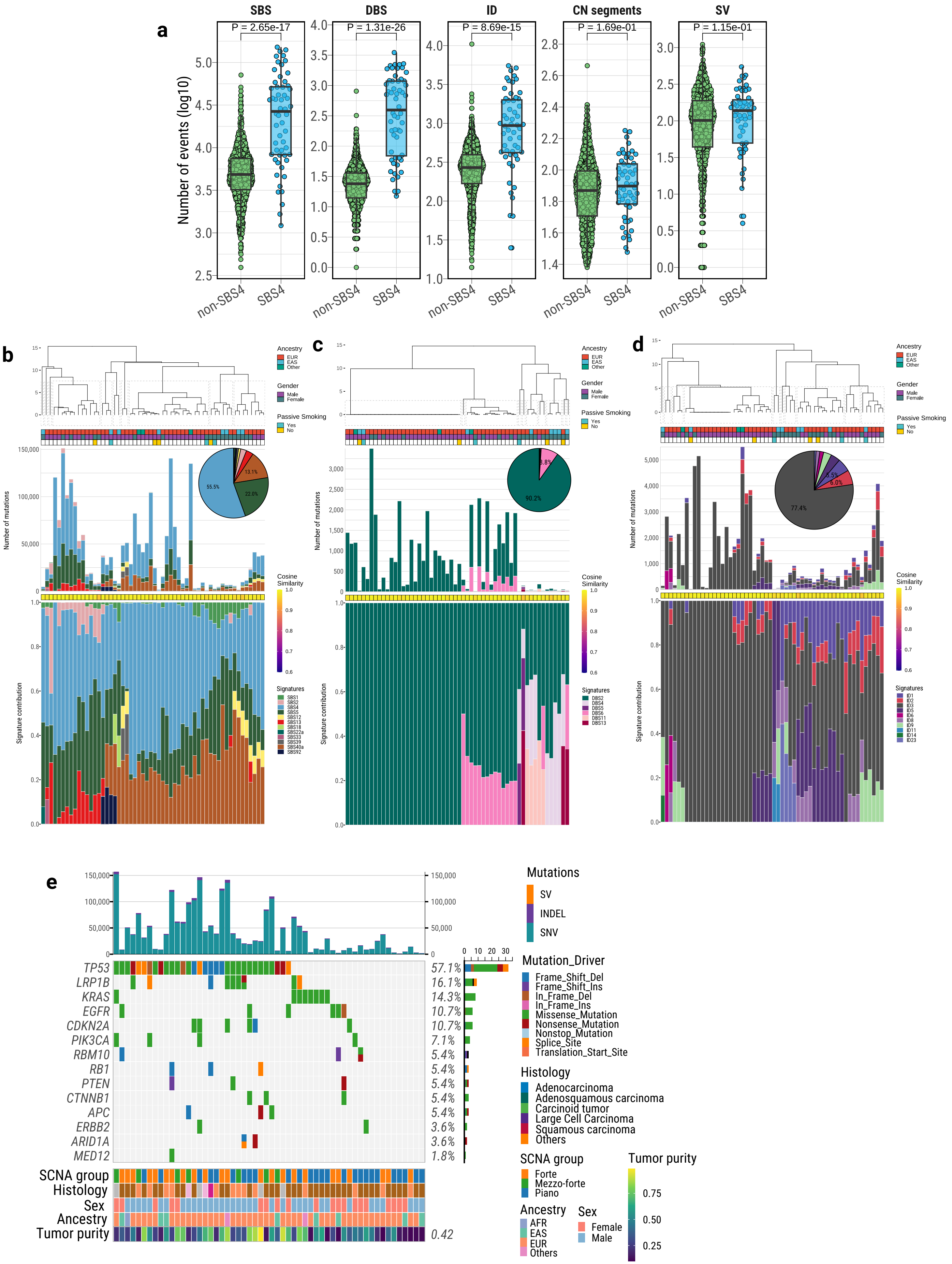

Extended Data Fig. 8

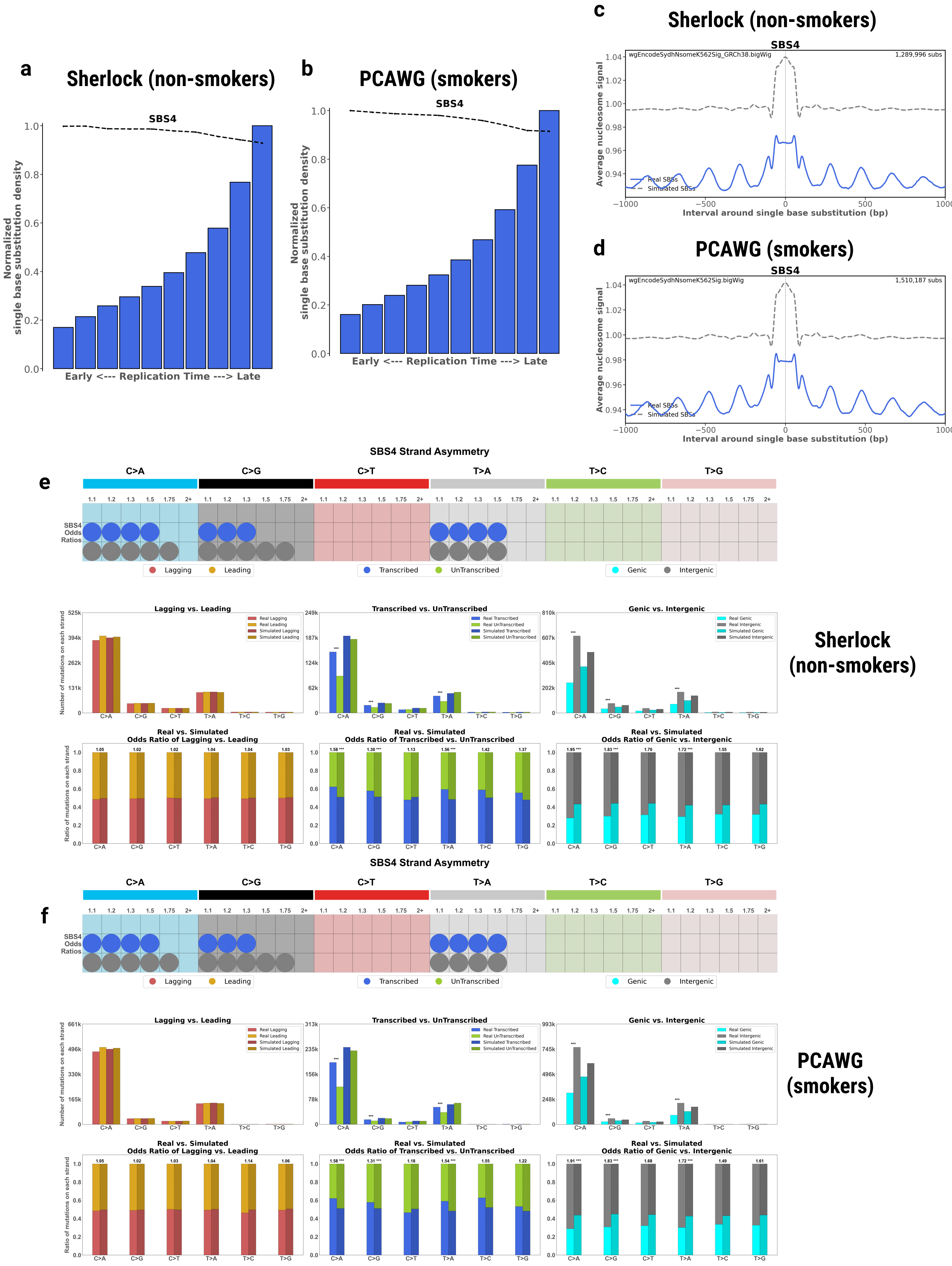

Extended Data Fig. 9

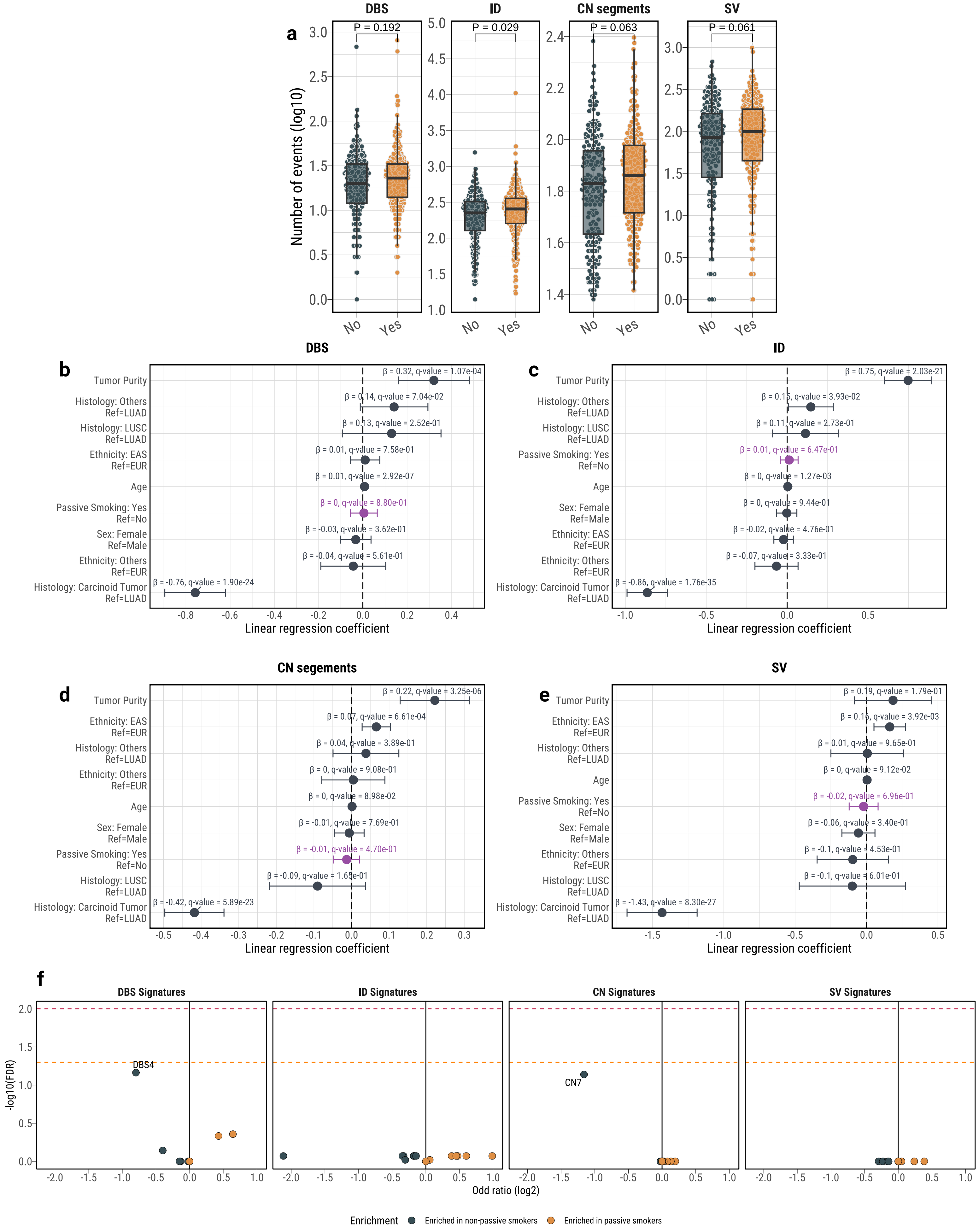

Extended Data Fig. 10

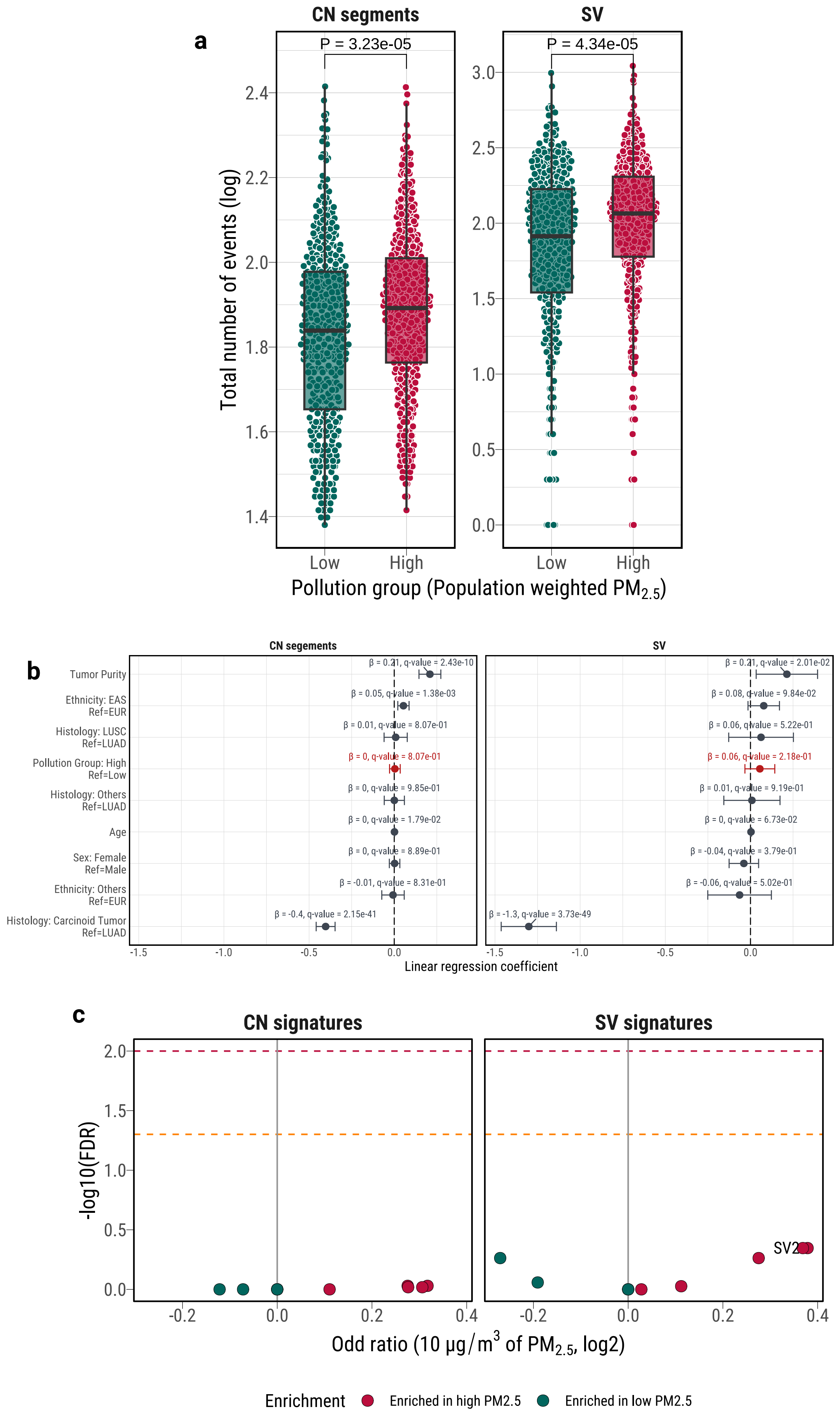

Extended Data Fig. 11

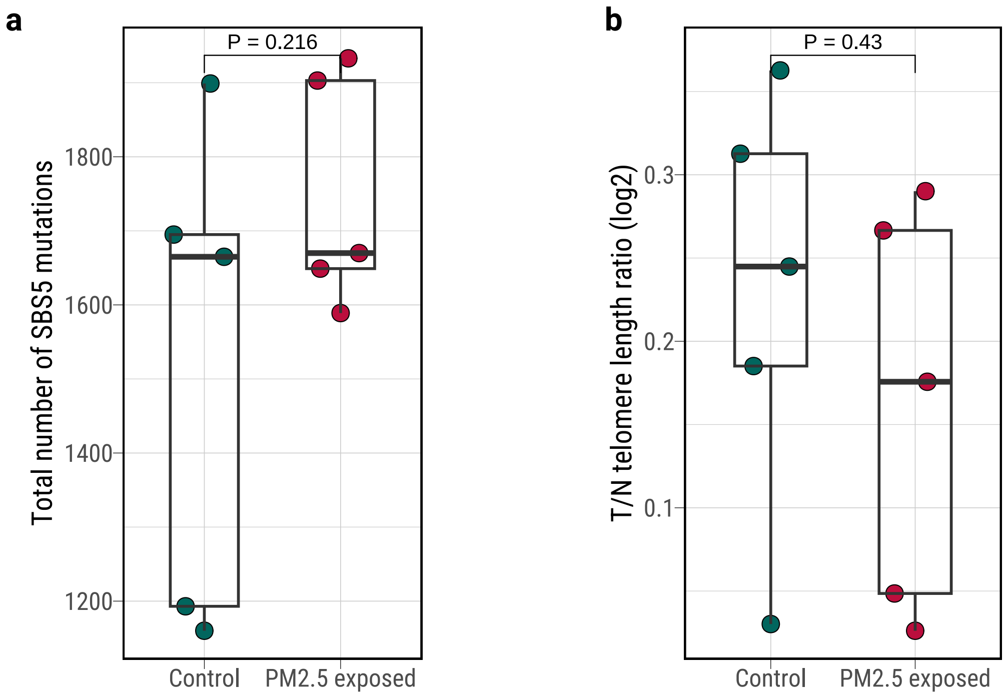

Extended Data Fig. 12

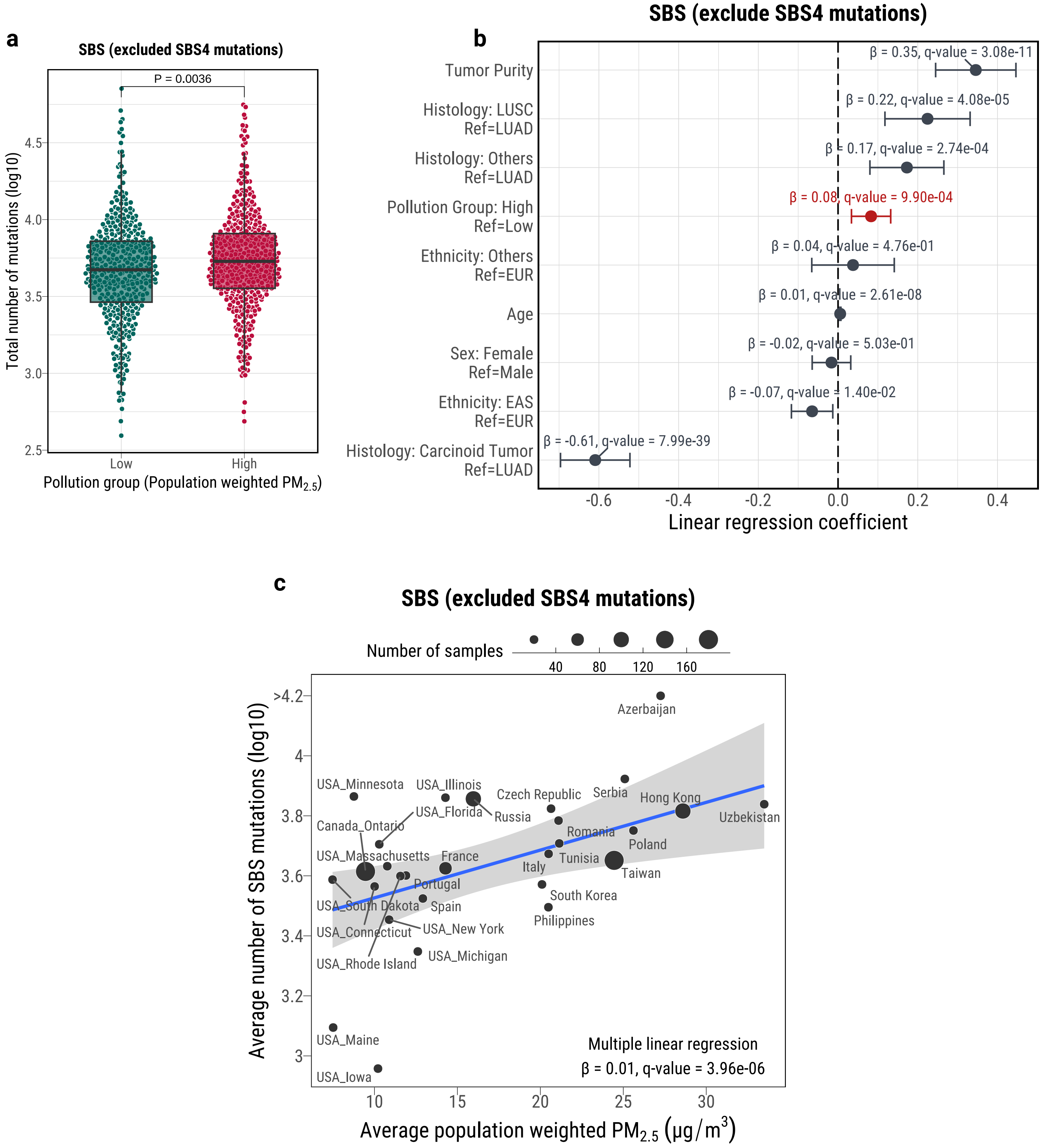

Supplement: Supplement 1 — Extended Data Fig. 1. Association of mutational signature prevalence and driver mutations with geographical regions, biological sexes, and EGFR mutation status in LCINS adenocarcinoma cases. a, DBS, ID, CN, and SV mutational signatures enrichment analysis with geographical regions. Horizontal lines marking statistically significant thresholds were included at 0.05 (dashed orange line) and 0.01 FDR value levels (dashed red line). Blue-colored signatures were enriched in North American and European patients, whereas red-colored signatures were enriched in East Asian patients. b, SBS, DBS, ID, CN, and SV mutational signatures enrichment analysis with biological sexes. Blue-colored signatures were enriched in males, whereas red-colored signatures were enriched in females. c-e, Detail of the enrichment of EGFR (c), TP53 (d), and KRAS (e) driver mutations in North American and European vs. East Asian LCINS adenocarcinoma cases. f, Driver mutations enrichment analysis with biological sexes. Blue-colored genes were enriched in males, whereas red-colored genes were enriched in females. h, SBS, DBS, ID, CN, and SV mutational signatures enrichment analysis with EGFR mutation status. Blue-colored signatures were enriched in EGFR mutant tumors, whereas red-colored signatures were enriched in EGFR wild-type tumors. Extended Data Fig. 2. Tumor mutational burden differences in LCINS across histologies. a-b, Quantification of tumor mutational burden according to different mutation types, including SBS, DBS, ID, number of copy number segments, and structural variants (a), as well as telomere length ratios between tumor and normal samples (b) across histologies. Extended Data Fig. 3. Repertoire of mutational signatures and driver mutations in LCINS carcinoids. a-b, Mutational signature landscape for SBS (a) and ID (b) mutation types, including absolute and relative number of mutations assigned to each mutational signature, unsupervised clustering based on the signature contributions, [file media-1.pdf]
