# Supplementary material for: The mutagenic forces shaping the genomic landscape of lung cancer in never smokers
Source: medRxiv. 2024 May 17:2024.05.15.24307318. Preprint. [Version 1] doi: 10.1101/2024.05.15.24307318 (PMC11118654; doi:10.1101/2024.05.15.24307318)

Supplementary Fig. 1

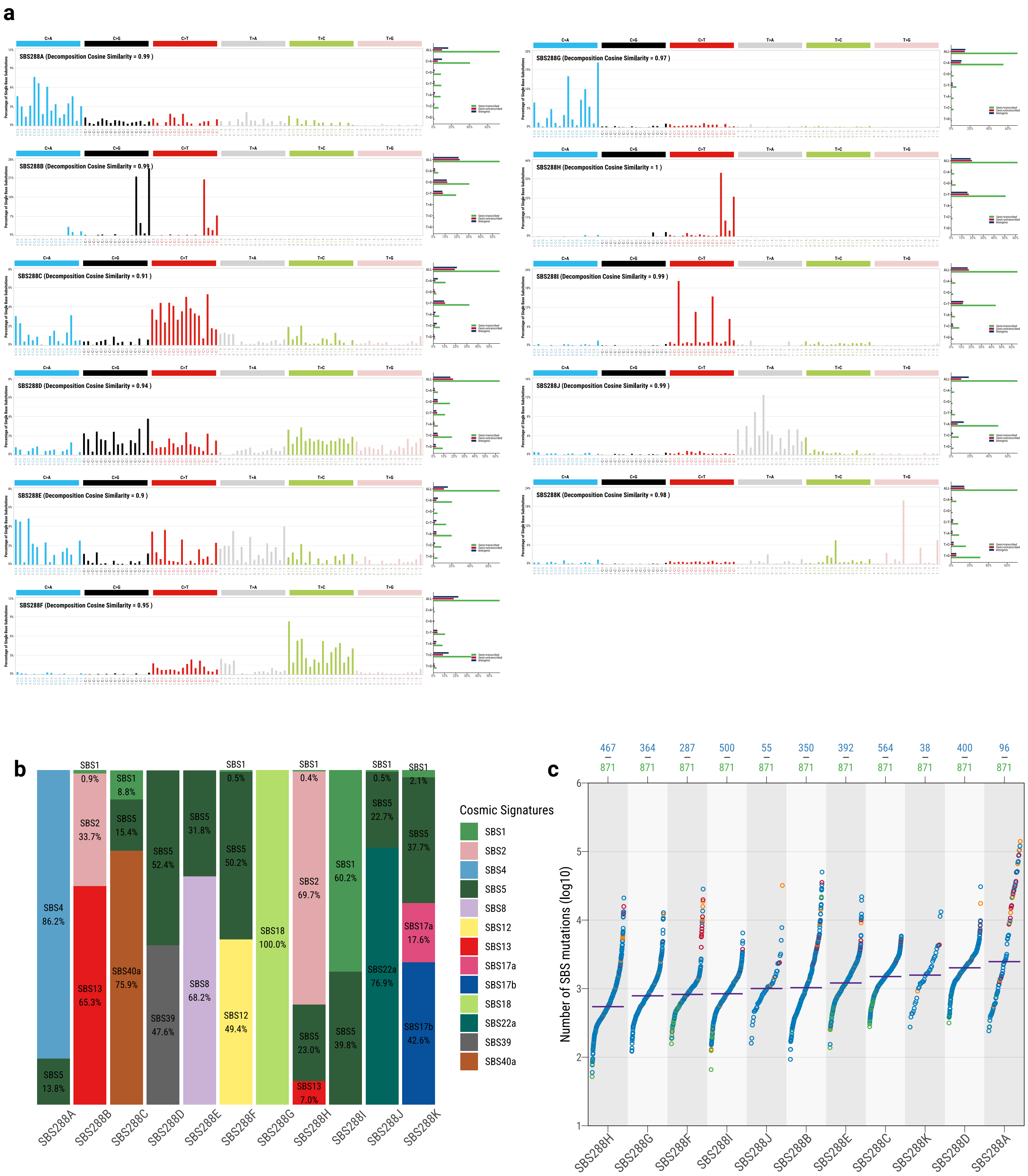

Supplementary Fig. 2

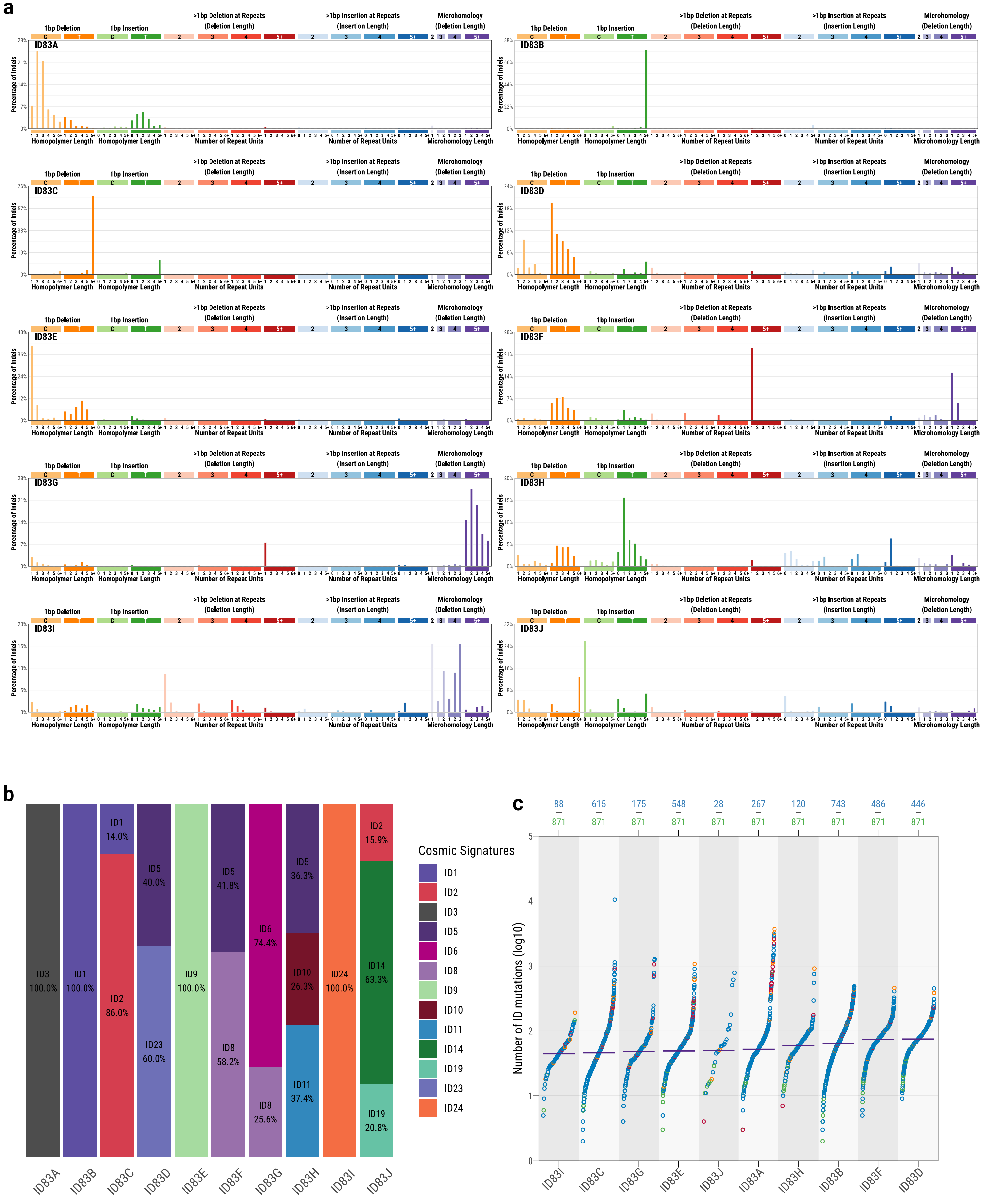

Supplementary Fig. 3

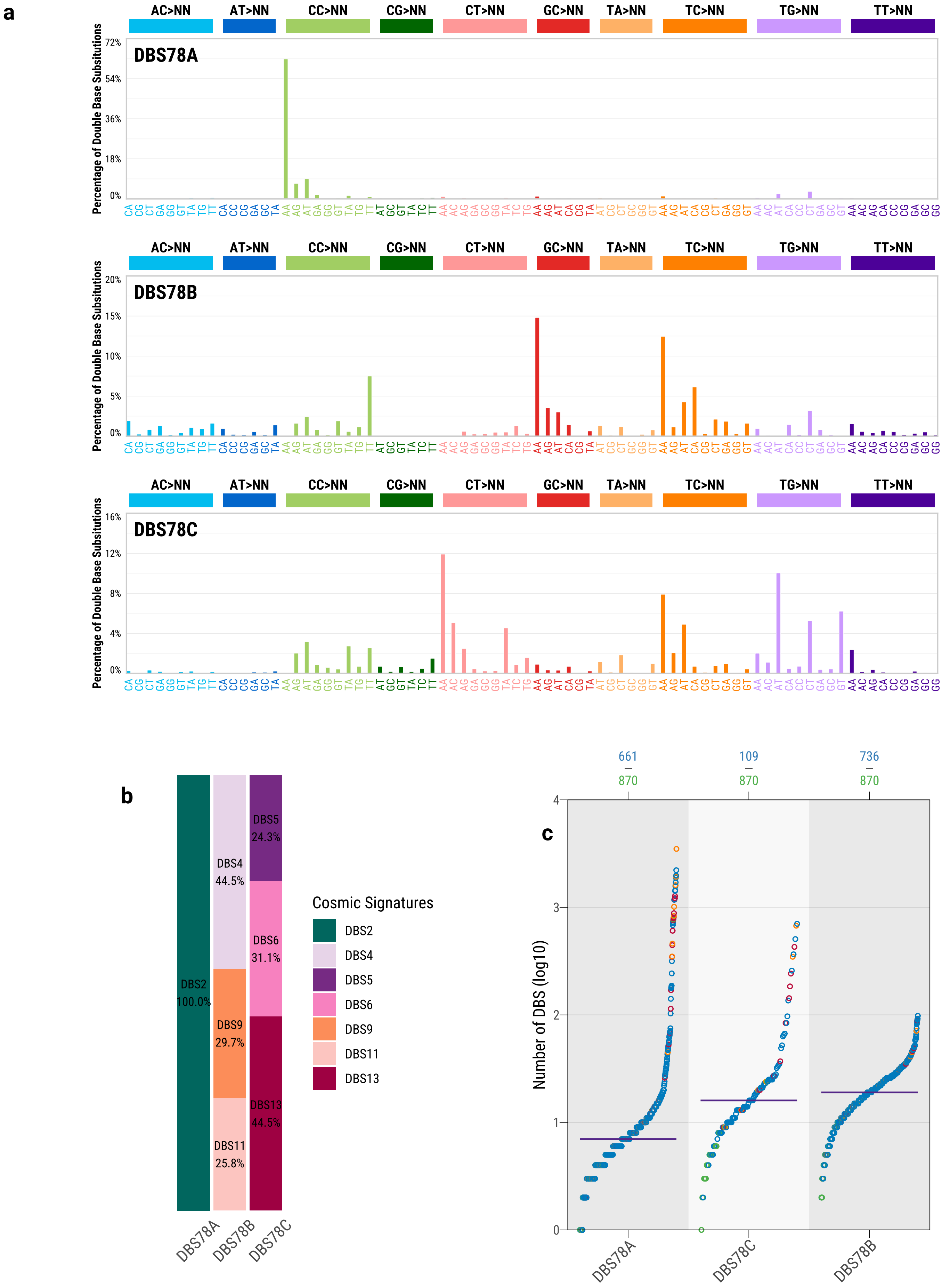

Supplementary Fig. 4

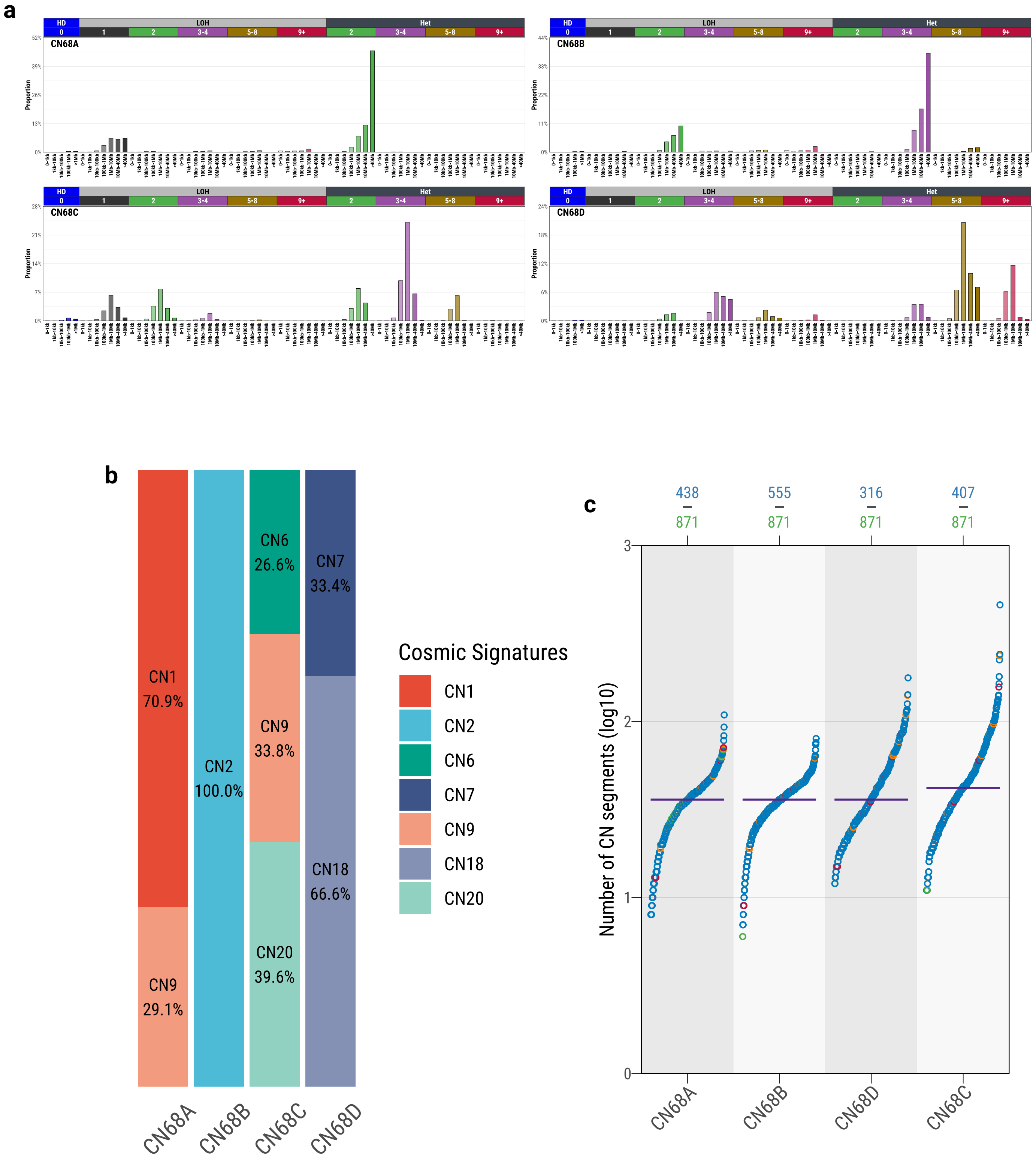

Supplementary Fig. 5

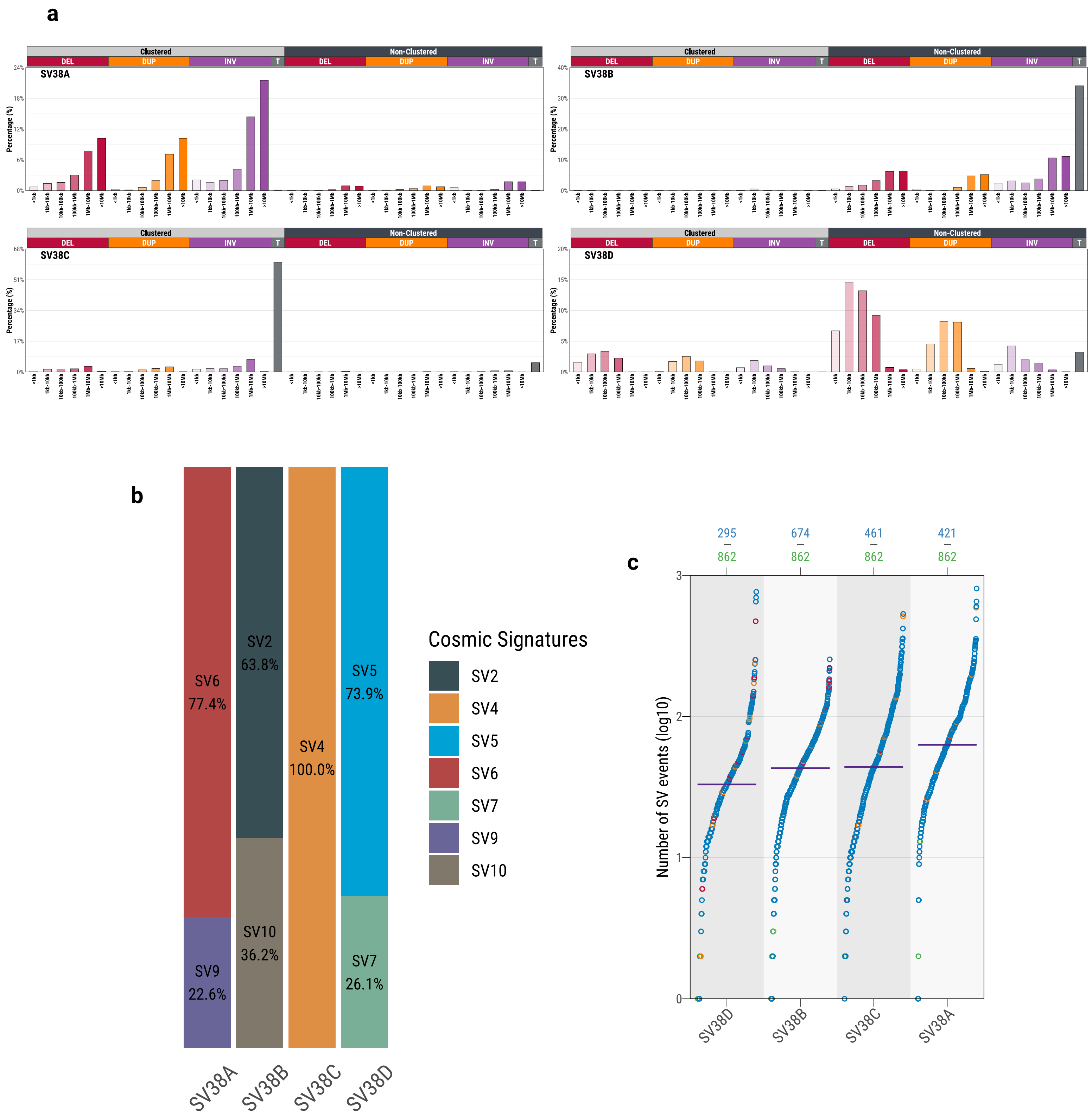

Supplementary Fig. 6

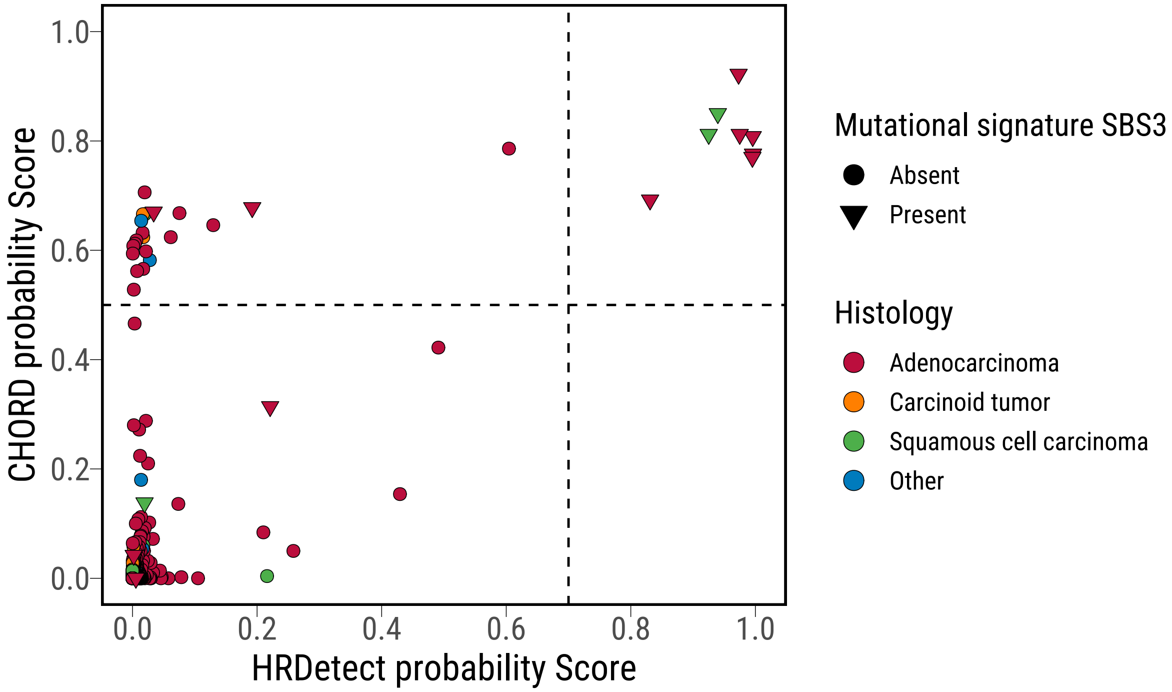

Supplementary Fig. 7

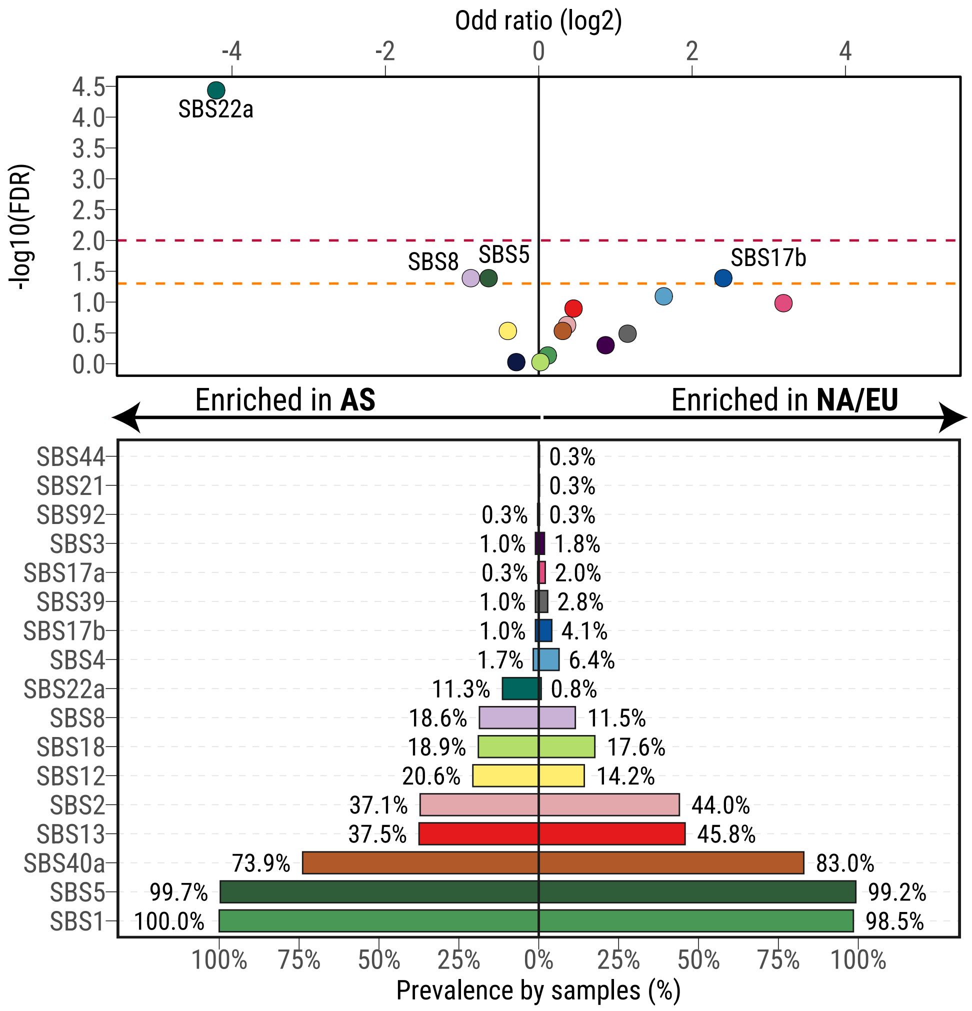

Supplementary Fig. 8

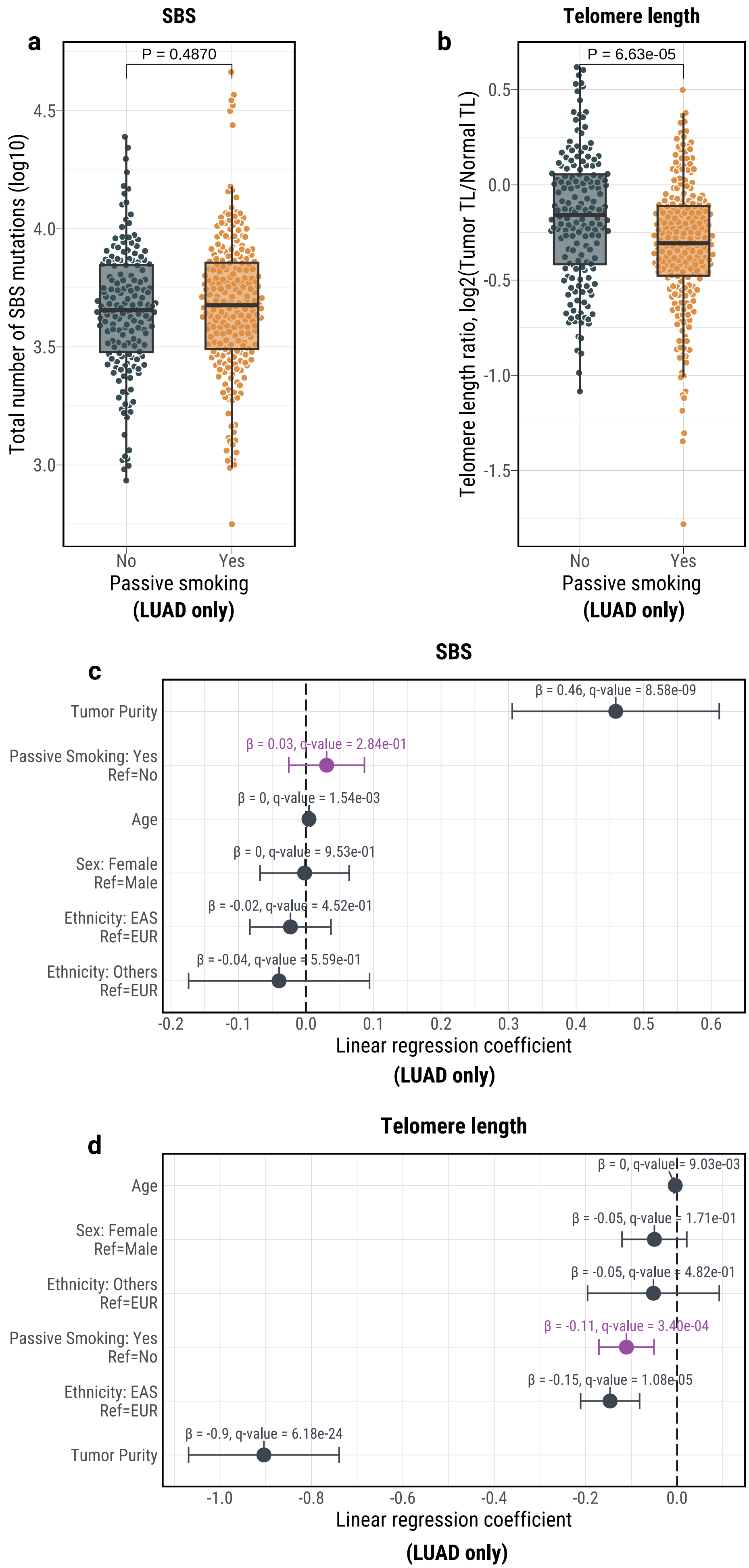

Supplementary Fig. 9

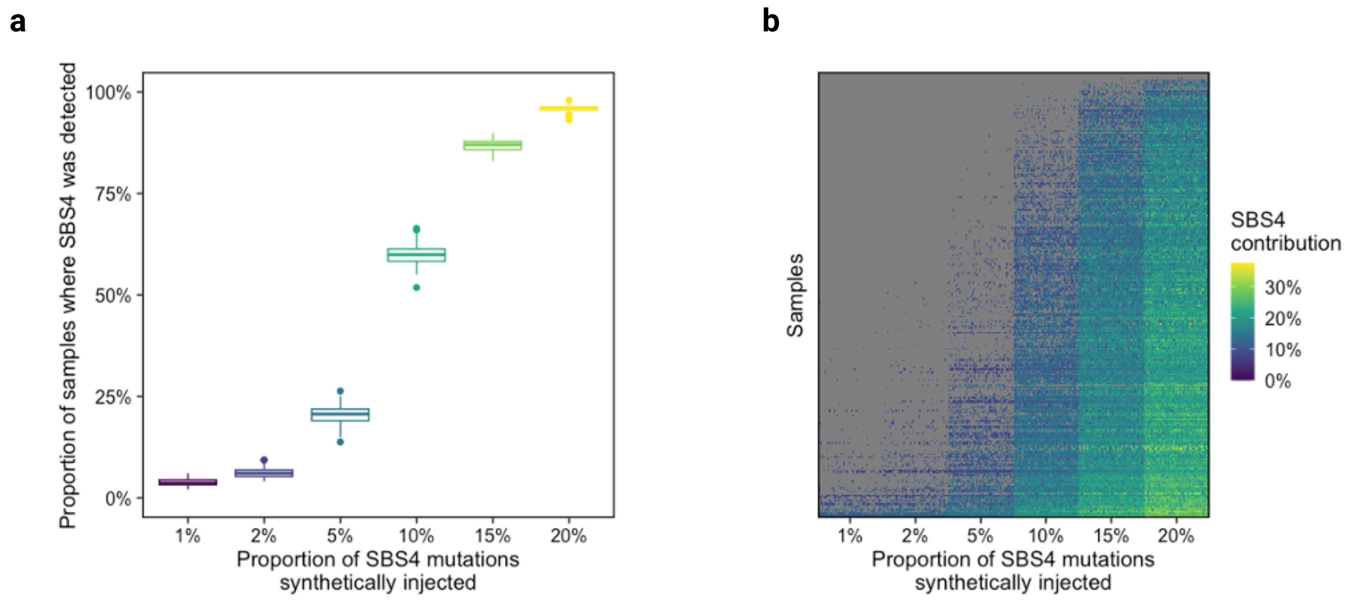

Supplementary Fig. 10

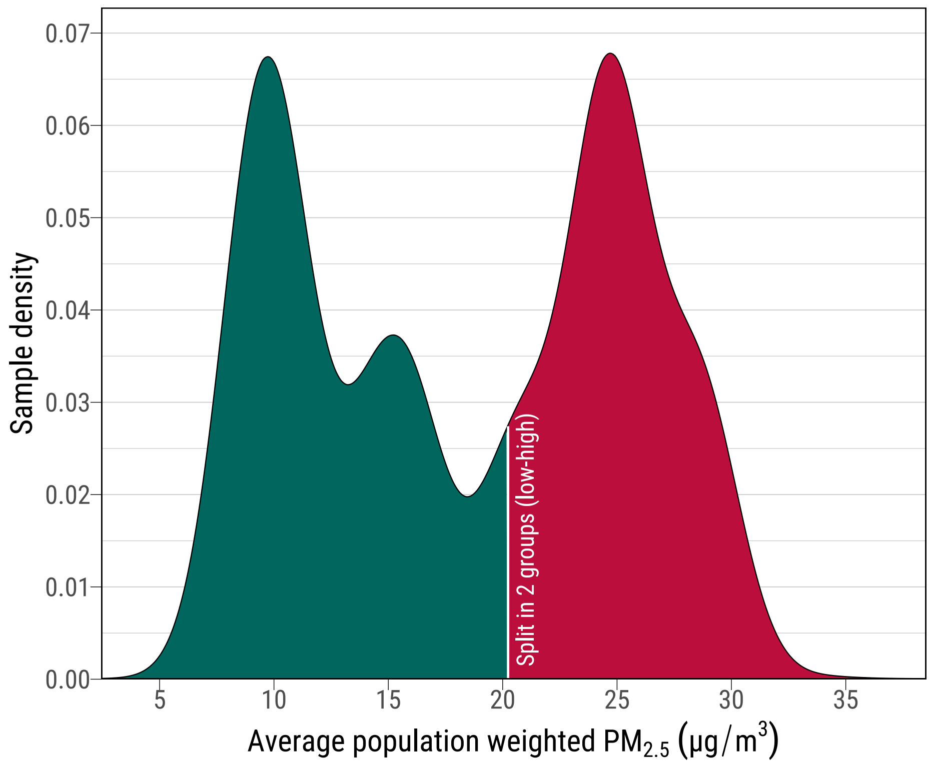

Supplement: Supplement 2 — Supplementary Fig. 1. De novo mutational signatures extracted using the SBS-288 mutational context. a, Mutational profiles of the de novo extracted signatures, with indication of the cosine similarity of the decomposition into COSMICv3.4 reference signatures. b, Contribution of the different COSMICv3.4 reference mutational signatures after decomposition of the de novo extracted signatures. c, Activity of de novo mutational signatures across samples, representing the total number of substitutions attributed to each signature in a given sample. Dots represent individual samples, colors different histology types, and purple horizontal bars median values across all histologies. The numbers on top indicate the total number of samples where a particular signature was found active (blue) and the total number of samples of the assessed cohort (green). Supplementary Fig. 2. De novo mutational signatures extracted using the ID-83 mutational context. a, Mutational profiles of the de novo extracted signatures. b, Contribution of the different COSMICv3.4 reference mutational signatures after decomposition of the de novo extracted signatures. c, Activity of de novo mutational signatures across samples, representing the total number of indels attributed to each signature in a given sample. Dots represent individual samples, colors represent different histology types, and purple horizontal bars represent median values across all histologies. The numbers on top indicate the total number of samples where a particular signature was found active (blue) and the total number of samples of the assessed cohort (green). Supplementary Fig. 3. De novo mutational signatures extracted using the DBS-78 mutational context. a, Mutational profiles of the de novo extracted signatures. b, Contribution of the different COSMIC v3.4 reference mutational signatures after decomposition of the de novo extracted signatures. c, Activity of de novo mutational signatures across samples, representing the total [file media-2.pdf]
